# Association between plasma ADAMTS-7 levels and ventricular remodeling in patients with acute myocardial infarction

**DOI:** 10.1186/s40001-015-0118-4

**Published:** 2015-03-21

**Authors:** Wenjing Wu, Yifeng Zhou, Yiyang Li, Jiahui Li, Yuannan Ke, Yong Wang, Jingang Zheng

**Affiliations:** Department of Cardiology, China-Japan Friendship Hospital, 2 Yinghua Dongjie, Beijing, 100029 China; Department of Gynaecology, The First Hospital of Jilin University, 71 Xinmin Street, Changchun, 130021 China

**Keywords:** Ventricular remodeling, Acute myocardial infarction, ADAMTS-7

## Abstract

**Background:**

The metalloproteinase family of a disintegrin and metalloproteinase with thrombospondin motifs (ADAMTS) degrades extracellular matrix. However, the relevance of the ADAMTS family to cardiovascular diseases remains largely unknown. The study aimed to examine plasma ADAMTS-7 levels in patients with acute myocardial infarction (AMI) and the relationship between plasma ADAMTS-7 levels and heart function.

**Methods:**

This was a prospective study performed in 84 patients with ST-elevation myocardial infarction (STEMI), 70 patients with non-STEMI (NSTEMI), and 38 controls. Enzyme-linked immunosorbent assay (ELISA) was used to measure plasma ADAMTS-7 levels. Cardiac structure and function were assessed using two-dimensional transthoracic echocardiography. Patients were stratified according to left ventricular ejection fraction (LVEF) ≤35% or >35%.

**Results:**

Plasma ADAMTS-7 levels were higher in patients with LVEF ≤35% compared with those with LVEF >35% (6.73 ± 2.47 vs. 3.22 ± 2.05 ng/ml, *P* < 0.05). Plasma ADAMTS-7 levels were positively correlated with brain natriuretic peptide (BNP), left ventricular mass index (LVMI), left ventricular end-diastolic diameter (LVEDD), and left ventricular end-systolic diameter (LVESD) and negatively correlated with the 6-min walk test (*P* < 0.05). According to the receiver operating characteristic (ROC) curve, using a cutoff value of plasma ADAMTS-7 of 5.69 ng/ml was associated with a specificity of 61.0% and a sensitivity of 87.6% for the diagnosis of heart failure after AMI. Logistic regression analysis indicated that the association between ADAMTS-7 and heart failure after AMI was independent from traditional cardiovascular risk factors and other biomarkers (odds ratio = 1.236, 95% confidence interval: 1.023 to 1.378, *P* = 0.021).

**Conclusions:**

Elevated ADAMTS-7 level may be involved in ventricular remodeling after AMI.

## Background

An increasing number of patients are suffering from heart failure after acute myocardial infarction (AMI), which is associated with high morbidity and mortality [1,2]. Ventricular remodeling plays an essential role in the development of heart failure, and the extracellular matrix (ECM) plays a major role in the maintenance of ventricular shape, size, and function [3]. After AMI, very early degradation of ECM causes progressive dilation and thinning in the infarction zone, contributing to infarct expansion and leading to severe consequences including left ventricular (LV) rupture, dilation, and dysfunction [4]. In addition, subsequent ECM disruption in the non-infarction zone leads to progressive global LV dilation over weeks [4].

ECM degradation requires the activation of extracellular proteases, which in turn facilitates ventricular remodeling [5]. Matrix metalloproteinases (MMP) play an important role in matrix degradation and ventricular remodeling [6,7]. MMP activation and consequent fibrillar collagen breakdown are responsible for adverse ventricular remodeling [8]. However, broad-spectrum MMP inhibitors led to severe side effects in clinical trials, indicating that more comprehensive knowledge about proteinases is needed [9].

Members of the recently identified metalloproteinase family of a disintegrin and metalloproteinase with thrombospondin motifs (ADAMTS) also degrade ECM. However, the relevance of the ADAMTS family to cardiovascular disease remains largely unknown [10]. ADAMTS-7 degrades cartilage oligomeric matrix proteins (COMP) found in the heart [11] and has been involved in inflammatory arthritis and bone growth [10,12]. Recently, Wang *et al*. [13] reported that ADAMTS-7 was a novel proteolytic culprit in vascular remodeling by mediating vascular smooth muscle cell migration and neointima formation in balloon-injured rat arteries [14], as well as in neointimal thickening after cardiac injury [15]. Du *et al*. [16] reported that upregulation of ADAMTS-7 by miR-29 repression mediated vascular smooth muscle calcification. ADAMTS-7 is involved in the proliferative response to vascular injury in a way that is similar to the progressive phase of atherosclerosis [17].

Therefore, we hypothesized that ADAMTS-7 participates in ventricular remodeling mediated, at least in part, by ADAMTS-7-dependent ECM degradation. In this pilot study, we selected patients with AMI (both ST-elevation myocardial infarction (STEMI) and non-STEMI (NSTEMI)) to detect plasma ADAMTS-7 levels and to explore the association between heart function and plasma ADAMTS-7.

## Methods

### Patients

Patients with AMI hospitalized between December 2012 and December 2013 at the cardiology department of the China-Japan Friendship Hospital were recruited, including 84 patients with STEMI and 70 patients with NSTEMI. Thirty-eight controls were also recruited from patients who underwent angiography and who subsequently showed no evidence of AMI. The diagnostic criteria for STEMI and NSTEMI were based on the 2013 ACCF/AHA guidelines for the management of STEMI [18], and the 2011 ESC guidelines for the management of acute coronary syndrome without ST-segment elevation [19].

For patients with STEMI or NSTEMI, inclusion criteria were as follows: 1) positive for troponin I; 2) history of chest pain lasting ≥20 min, no response to nitroglycerine or ECG changes (STEMI: ST-segment elevation measured at J-point in two continuous leads or with new onset of left bundle branch block; NSTEMI: ST-segment shifts and T-wave changes); 3) patient presented at the hospital within 24 h of symptoms onset; 4) patient consented to the study and blood was sampled for ADAMTS-7 measurement before any intervention; and 5) signed the consent form. For controls, inclusion criteria were as follows: 1) complaints of ischemic pains; 2) angiography showed vascular stenosis <50%; 3) patient consented to the study, and blood was sampled for ADAMTS-7 measurement before any intervention; and 4) signed the consent form. For all subjects, exclusion criteria were as follows: 1) severe hepatic or renal insufficiency; 2) rheumatologic disorders; 3) malignancy; or 4) active infection.

The study protocol was approved by the research ethics committee of the China-Japan Friendship Hospital and all patients provided a written informed consent.

### Data collection

Gender, age, body mass index (BMI), medical history, and results of laboratory examinations were collected from the hospital computer system.

### Sample collection

Six-milliliter samples of whole blood were collected into EDTA anticoagulant tubes and were stored at 4°C. Samples were centrifuged for 15 min at 1,000 × *g* at 4°C within 30 min of collection. Samples were stored at −80°C until analysis.

### Plasma ADAMTS-7 levels and biochemical assays

Plasma ADAMTS-7 levels were measured using an enzyme-linked immunosorbent assay (ELISA) for human ADAMTS-7 (Human ADAMTS-7 ELISA Kit; MyBioSource Inc., San Diego, CA, USA). Optical density was measured at 450 nm. The lowest detectable limit of ADAMTS-7 concentration was 1.529 ng/mL, according to the manufacturer’s instructions, and the coefficient of variation was <10%. Brain natriuretic peptide (BNP), serum lipids, high-sensitivity C-reactive protein (hs-CRP), fasting blood glucose, and serum creatinine were measured using standard clinical methods at the China-Japan Friendship Hospital using a Hitachi 7600 series automatic analyzer (Hitachi, Tokyo, Japan).

### Echocardiography

Cardiac structure and function were assessed using two-dimensional transthoracic echocardiography performed and interpreted by cardiologists specialized in echocardiography and who were blind to the patient’s clinical status. Echocardiography was performed using a Vivid E9 Echopac system with a high-definition M5S probe (GE Healthcare, Waukesha, WI, USA). Gain settings and pulse repetition frequency were adjusted to optimize color saturation and to avoid aliasing. LV ejection fraction (LVEF) was calculated using the biplane Simpson method. LV end diastolic/systolic diameter (LVEDD/LVESD) and interventricular septum thickness (IVST) were measured and recorded [20]. The LV mass (LVM) was derived using the following formula: LVM (g) = 0.8 × (1.04 × (IVST + LVEDD + LVPWT)^3^ − LVEDD^3^) + 0.6 [21], where LVPWT is the abbreviation for LV posterior wall thickness. LVM was subsequently adjusted for body surface area (BSA) to obtain the LVM index (LVMI) value: LVMI (g/m^2^) = LVM/BSA.

Patients with STEMI or NSTEMI were divided into two groups according to a LVEF ≤35% or >35%.

### Six-min walk test

The 6-min walk test was conducted in an enclosed 30-m-long corridor. The patients were instructed to walk from end to end, covering as much ground as they could during the 6 min test. The supervisor sat in a chair at one end of the course and delivered a predetermined set of encouraging phrases such as ‘You are doing well’ or ‘Keep up the good work.’ The total distance was recorded.

### Statistical analysis

SPSS 19.0 (IBM, Armonk, NY, USA) and Prism 5.0 (GraphPad Software Inc., San Diego, CA, USA) were used for data analysis. Descriptive data are presented as means ± standard deviation (SD) for normally distributed variables or as medians (interquartile range) for non-normally distributed variables and as frequencies for categorical variables. Comparisons were performed using independent *t*-tests (normally distributed), Mann–Whitney *U*-tests (non-normally distributed), or chi-squared tests (categorical data). Receiver operating characteristic (ROC) curve was used to determine specificity and sensitivity for predicting heart failure after AMI. Linear associations were assessed using the Kendall’s tau-b analysis. Binary logistic regression was used to identify the predictive impacts of plasma ADAMTS-7 on heart failure. The odd ratios (OR) and the corresponding 95% confidence intervals (95% CI) were calculated. Two-sided *P* values of <0.05 were considered significant.

## Results

### Patient characteristics

Age and frequency of hypertension, diabetes, and male gender were higher in patients with AMI compared with controls (*P* < 0.05). The proportion of patients with serum creatinine >140 μmol/l was significantly higher in patients with AMI. There were no differences in BMI, blood glucose, and blood lipids between groups (Table 1). Levels of high-sensitivity C-reactive protein (hsCRP) were elevated in patients with AMI compared with controls (*P* < 0.05).

**Table 1:** **Characteristics of the patients**

**Characteristics**	**LVEF ≤35% (** ***n*** **= 42)**	**LVEF >35% (** ***n*** **= 112)**	**Controls (** ***n*** **= 38)**	***P*** **value** ^**b**^
Age, years	66.5 ± 12.4	68.7 ± 10.4	56.6 ± 11.5	<0.05
Gender, male/female	28/14 (66.7/33.3%)	71/41 (63.4/36.6%)	21/17 (55.6%/44.7%)	<0.05
BMI (kg/m^2^)	24.4 ± 3.0	24.7 ± 3.8	26.1 ± 4.1	0.23
History of smoking, *n* (%)	12 (28.6)	30 (26.5)	11 (28.9)	0.42
Serum creatinine >140 μmol/L, *n* (%)	13 (30.95)^a^	6 (5.36)^a^	0 (0.00)	<0.05
History of hypertension, *n* (%)	28 (66.7)	73 (65.2)	16 (42.1)	<0.05
History of diabetes, *n* (%)	19 (45.2)	54 (48.2)	9 (23.7)	<0.05
Glucose (mmol/L)	8.17 ± 3.86	8.42 ± 3.56	5.64 ± 1.21	0.23
Total cholesterol (mmol/L)	4.64 ± 0.85	4.36 ± 1.17	4.66 ± 0.89	0.90
Triglycerides (mmol/L)	1.59 ± 1.21	1.38 ± 0.72	1.88 ± 1.34	0.08
HDL-cholesterol (mmol/L)	1.07 ± 0.33	1.12 ± 0.29	1.06 ± 0.32	0.06
LDL-cholesterol (mmol/L)	2.83 ± 0.84	2.72 ± 0.94	2.76 ± 0.64	0.97
hsCRP (mg/L)	12.32 ± 53.46	11.68 ± 38.48	4.89 ± 9.30	<0.05
Type of AMI, *n* (%)				<0.05
STEMI	33 (78.6)	51 (45.5)		
NSTEMI	9 (21.4)	61 (54.5)		
BNP	1,670.71 ± 1,074.44^a^	433.72 ± 285.67^a^	60.69 ± 36.98	<0.05
LVEF	28.43 ± 4.79^a^	47.31 ± 7.69^a^	57.42 ± 6.28	<0.05
LVMI	90.51 ± 14.39^a^	70.43 ± 12.04^a^	82.67 ± 20.05	<0.05
LVEDD	61.95 ± 4.80^a^	49.45 ± 4.72^a^	50.23 ± 5.98	<0.05
LVESD	38.05 ± 5.73^a^	31.37 ± 4.08^a^	30.37 ± 4.15	<0.05
Six-min walk test	171.07 ± 70.32^a^	465.13 ± 189.00^a^		<0.05
Plasma ADAMTS-7 levels	6.73 ± 2.47^a^	3.22 ± 2.05^a^	1.63 ± 1.58	<0.05

^a^Mean *P* < 0.05 compared between the LVEF ≤35 group and LVEF >35% group; ^b^mean comparison between the three groups. ADAMTS, a disintegrin and metalloproteinase with thrombospondin motifs; AMI, acute myocardial infarction; BMI, body mass index; HDL, high-density lipoprotein; LDL, low-density lipoprotein; hsCRP, high-sensitivity C-reactive protein; STEMI, ST-elevation myocardial infarction; NSTEMI, non-ST-elevation myocardial infarction; BNP, brain natriuretic peptide; LVEF, left ventricular ejection fraction; LVMI, left ventricular mass index; LVEDD, left ventricular end-diastolic diameter; LVESD, left ventricular end-systolic diameter.

### Plasma ADAMTS-7 in patients with AMI and LVEF ≤35% or LVEF >35%

Patients with AMI were divided according to LVEF ≤35% or >35%. Plasma levels of ADAMTS-7 were higher in patients with LVEF ≤35% compared with those with LVEF >35% (6.73 ± 2.47 vs. 3.22 ± 2.05 ng/ml, *P* < 0.05).

### Association between ADAMTS-7 levels and heart failure

The median ADAMTS-7 level among the 154 patients with AMI (84 STEMI patients and 70 NSTEMI patients) was 4.48 ng/ml (range: 0.34 to 14.26 ng/ml). Kendall’s tau-b analysis showed that plasma ADAMTS-7 levels were positively correlated with BNP (*r* = 0.732, *P* = 0.005), LVMI (*r* = 0.714, *P* = 0.02), LVEDD (*r* = 0.693, *P* = 0.04), and LVESD (*r* = 0.647, *P* = 0.03) and negatively correlated with the 6-min walk test (*r* = −0.653, *P* = 0.02) and LVEF (*r* = −0.624, *P* = 0.04) (Table 2).

**Table 2:** **Correlation between ADAMTS-7 levels and clinical parameters in patients with AMI**

**Variables**	**Correlation coefficient (** ***r*** **)**	***P*** **value**
BNP	0.732	0.005
LVMI	0.714	0.016
LVEDD	0.693	0.038
LVESD	0.647	0.026
Six-min walk test	−0.653	0.024
LVEF	−0.624	0.041

ADAMTS, a disintegrin and metalloproteinase with thrombospondin motifs; AMI, acute myocardial infarction; BNP, brain natriuretic peptide; LVEF, left ventricular ejection fraction; LVMI, left ventricular mass index; LVEDD, left ventricular end-diastolic diameter; LVESD, left ventricular end-systolic diameter.

**Figure 1:**
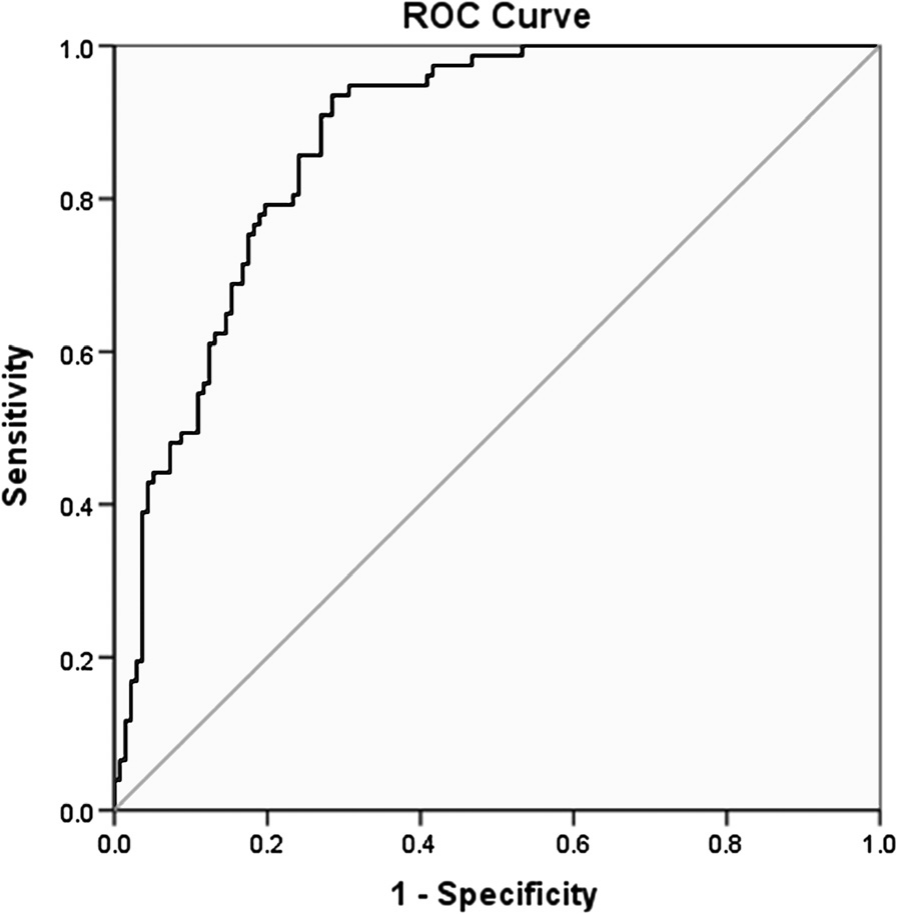
**ROC curve analysis of plasma ADAMTS-7 levels for the prediction of heart failure after AMI.** ROC, receiver operating characteristic.

According to the ROC curve, a cutoff value of plasma ADAMTS-7 of 5.68 ng/ml resulted in a specificity of 61.0% and a sensitivity of 87.6% for the diagnosis of heart failure after AMI (Figure 1).

To control for potential confounders, a multivariate analysis was performed. Age, gender, BMI, hypertension, hyperlipidemia, diabetes, blood glucose, total cholesterol, triglycerides, high-density lipoprotein (HDL)-cholesterol, low-density lipoprotein (LDL)-cholesterol, and hsCRP were set as independent variables, and heart failure was set as the dependent variable. Results indicated that elevated plasma ADAMTS-7 levels were independently associated with heart failure after AMI (OR = 1.236, 95% CI: 1.023 to 1.378, *P* = 0.021) as well as age (OR = 1.158, 95% CI: 1.013 to 1.438, *P* = 0.035) and hsCRP (OR = 1.285, 95% CI: 1.002 to 1.411, *P* = 0.016) (Table 3).

**Table 3:** **Multivariate analysis of factors associated with heart failure after AMI**

**Variables**	***P*** **value**	**Exp (B)**	**95% CI for Exp (B)**
Age	0.035	1.158	1.013 to 1.438
Gender	0.093	1.213	0.872 to 1.365
BMI	0.523	1.233	0.721 to 1.259
Hypertension history	0.269	1.000	0.963 to 1.101
Hyperlipidemia history	0.436	0.983	0.925 to 1.028
Diabetes history	0.377	0.797	0.740 to 1.029
hsCRP	0.016	1.285	1.002 to 1.411
Plasma ADAMTS-7	0.022	1.242	1.015 to 1.378

95% CI, 95% confidence interval; ADAMTS, a disintegrin and metalloproteinase with thrombospondin motifs; AMI, acute myocardial infarction; BMI, body mass index; hsCRP, high-sensitivity C-reactive protein.

## Discussion

The aim of the present study was to examine plasma ADAMTS-7 levels in patients with AMI and the relationship between plasma ADAMTS-7 levels and heart function. Results showed that plasma ADAMTS-7 levels were higher in patients with LVEF ≤35% compared with those who had a LVEF >35%. Plasma ADAMTS-7 levels were positively correlated with BNP, LVMI, LVEDD, and LVESD and negatively correlated with the 6-min walk test, which is consistent with the role of MMP in the progression of ventricular remodeling [22]. According to the ROC curve, using a cut-off value of plasma ADAMTS-7 of 5.69 ng/ml was associated with a specificity of 61.0% and a sensitivity of 87.6% for the diagnosis of heart failure after AMI. Logistic regression analysis indicated that the association between ADAMTS-7 and heart failure after AMI was independent of traditional cardiovascular risk factors and other biomarkers.

The early induction of the enzymatic activity of ECM-degrading proteases precedes ventricular remodeling. From the therapeutic standpoint, inhibition of such proteases would be beneficial for the treatment of patients with heart failure. Given the disappointing side effects of broad-spectrum MMP inhibitors, more comprehensive knowledge about proteinases is needed [9]. Thus, unraveling the role of individual proteases in cardiovascular disease is of particular importance. To the best of our knowledge, the present study is the first to show an association between plasma ADAMTS-7 levels and LV function after AMI. Indeed, low LVEF after AMI is indicative of more extensive cardiac damage and is associated with a bad prognosis [23]. However, the exact implication of ADAMTS-7 in ventricular remodeling still needs to be exactly determined.

Unlike other metalloproteinases, ADAMTS demonstrates a narrow substrate specificity due to the various exosites located in the C-terminal regions of the enzymes, which influence protein recognition and matrix location [24], suggesting that ADAMTS could potentially be safe pharmaceutical targets [13]. ADAMTS-7 expression was detected as a 5-kb transcript in adult human samples, with the heart, pancreas, kidney, skeletal muscle, and liver having the highest levels of expression [13]. Previous studies demonstrated that ADAMTS-7 facilitates vascular muscle cell migration and intimal thickening after vascular injury and plays an important role in atherosclerosis and restenosis by degrading COMP [13-16]. Huang *et al*. [25] reported the presence of COMP in cardiomyocytes, where it plays an essential role during the initiation and progression of dilated cardiomyopathy. Therefore, it may be hypothesized that ADAMTS-7 affects the ventricular remodeling process by degrading COMP in patients with AMI. However, it is possible that ADAMTS-7 degrades other substrates. Nevertheless, a study showed an association between the ADAMTS-7 locus and angiographic coronary artery disease [15]. Further studies are necessary to assess the mechanisms of ADAMTS-7 in ventricular remodeling and heart diseases. Other members of the ADAMTS family also warrant investigation in AMI [26].

The present study is not without limitations. This was a single-center study with a relatively small sample size. For now, the cutoff value is purely suggestive since there is no established reference available from large clinical trials. Finally, the present study was not designed to provide any mechanistic insights into the roles of ADAMTS-7 in patients with heart failure after AMI. Larger multi-center trials are necessary to determine adequately the role of ADAMTS-7 in heart failure after AMI.

## Conclusions

In conclusion, elevated ADAMTS-7 levels may be involved in ventricular remodeling after AMI. High ADAMTS-7 levels were independently associated with poor prognosis in patients with AMI.

## Abbreviations

95% CI: 95% confidence intervals
AMI: acute myocardial infarction
BMI: body mass index
BNP: brain natriuretic peptide
COMP: cartilage oligomeric matrix proteins
ECM: extracellular matrix
ELISA: enzyme-linked immunosorbent assay
HDL: high-density lipoprotein
hs-CRP: high-sensitivity C-reactive protein
IVST: interventricular septum thickness
LDL: low-density lipoprotein
LV: left ventricular
LVEDD/LVESD: left ventricular end diastolic/systolic diameter
LVEF: left ventricular ejection fraction
LVM: left ventricular mass
LVMI: left ventricular mass index
MMP: matrix metalloproteinase
NSTEMI: non-ST-elevated myocardial infarction
OR: odds ratio
ROC: receiver operating characteristic
STEMI: ST-elevated myocardial infarction
